# Digital decision support integrated with diagnostics and precision fungicide application for Southern Corn Leaf Blight in maize

**DOI:** 10.1038/s41598-026-38151-0

**Published:** 2026-02-11

**Authors:** G. Jadesha, Anurag Dhole, D. Deepak, Manjunath Hubballi

**Affiliations:** 1 College of Agriculture, GKVK, University of Agricultural Sciences, Bangalore, 560065 Karnataka India; 2 Department of Mechatronics, Manipal Institute of Technology, Manipal Academy of Higher Education, Manipal, 576104 Karnataka India; 3 Directorate of Research, UHS, Bagalkot, 587104 Karnataka India

**Keywords:** Southern Corn Leaf Blight, Deep-learning, VGG16, Decision support system, Precision agriculture, Computational biology and bioinformatics, Plant sciences

## Abstract

Southern Corn Leaf Blight (SCLB, also called Maize Leaf Blight, MLB), caused by *Bipolaris maydis* (teleomorph: *Cochliobolus heterostrophus*), severely limits maize yield under favourable conditions. Rapid detection and precise interventions are essential for sustainable production. We present an AI-driven framework integrating deep learning diagnostics, precision fungicide application, and a digital decision support system (DSS) for field-level SCLB management. Thirteen machine learning (ML) and deep learning (DL) algorithms were evaluated, with VGG16 achieving the highest performance (accuracy 97.0%, precision 0.98, recall 0.96, F1-score ≥ 0.97, AUC-ROC = 1.00). Feature extraction analysis highlighted VGG16’s ability to capture hierarchical disease-specific patterns (score = 0.95), and error- and variance-based assessment confirmed minimal prediction errors (MAE = 0.06, RMSE = 0.16, Explained Variance = 0.90, MBD = − 0.02). Confusion matrix analysis revealed only a small number of misclassifications (4 false negatives and 9 false positives), demonstrating excellent generalization. Grad-CAM heatmaps, t-SNE visualization, and learning curves confirmed lesion-focused predictions and feature separability. Two-year field trials (2023 and 2024) validated precision fungicide application (Azoxystrobin 18.2% + Difenoconazole 11.4% SC), reducing disease severity to ≈ 10% PDI (86.2% reduction) and increasing grain yield to 83.7 q/ha (C: B ratio 1:2.41). The Streamlit-based DSS provides actionable, real-time advisories, offering a scalable AI platform for automated disease detection and precision agriculture in maize. The proposed framework can be extended to other foliar diseases and integrated with IoT-based sensing for region-wide advisory systems.

**Supplementary Information:**

The online version contains supplementary material available at 10.1038/s41598-026-38151-0.

## Introduction

Maize (*Zea mays* L.), one of the most important cereal crops in the world, is a crop consumed as food and feed for animals, while also being used to make biofuel and industrial products^1^. Its international significance in relation to food security and economic stability, makes it extremely susceptible to yield losses due to biotic stresses and one of these, Southern Corn Leaf Bight (SCLB) caused by *Bipolaris maydis* (teleomorph: *Cochliobolus heterostrophus*), is particularly destructive. The US-corn epidemic in 1970-71 decreased maize yield by 15%, causing losses of more than US$1.0 billion (more than > US$6.0 billion, in today’s value) at the time^2^. These historical lessons reinforce the imperative of rapid and trustworthy approaches for SCLB detection and management options. Traditional scouting techniques are, however, time-consuming, subjective and not capable of providing real-time information. These constraints have increased the attention in use of advanced techniques such as artificial intelligence (AI), machine-learning (ML) and deep-learning (DL) that show great potential for plant health monitoring practice (Jadesha et al.^3^,.

### Advances in artificial intelligence (AI) for plant disease detection

Recent progress in AI, has led to transformational changes in plant disease diagnosis through the provision of robust, scalable field-ready solutions. Convolutional Neural Networks (CNNs)-based image systems are capable of capturing fine-grained visual patterns, allowing accurate classification of plant diseases^4^. Vision Transformers generalize these features using self-attention mechanisms and are capable of fine-grained feature extraction in presence of various field conditions^5^. Among CNNs model, VGG16 is proving to be performing well with accuracies exceeding 95% on various plant diseases dataset^6^. Transfer learning is another effective way to improve generalizability and robustness, in combination with regularization techniques such as dropout and batch normalization, particularly in data impoverished settings^7–9^. Together, these advancements illustrate the transformative effect AI will have on precision agriculture and sustainable crop protection^10^; Jadesha et al.^3^,.

### Role of deep-learning in disease detection

Deep-learning has further accelerated progress by outperforms classical ML in disease detection. Compared to traditional algorithms which are labour-intensive due to manual feature engineering, DL architectures can directly infer the informative representations from raw images, which has potential to detect more sophisticated and subtle disease patterns^11,12^. Specifically, CNNs, including VGG16, are suitable for large scale high dimensional datasets while being robust to environmental variability including lighting and the background noise as well as symptom heterogeneity^13,14^. Reported accuracies higher than 95% in various researches asserted the superiority of DL over ML based methods (Alatawi et al., 2022; Bhagwat & Dandawate^6^,. Among DL models, the transfer learning with VGG16 has had great influence. Using knowledge learned from big image datasets pretrained VGG16 extracts hierarchical visual representation features, and obtained the state-of-the-arts with better generalization capability compared with some traditional methods on small-scale dataset^9,15^. Hybrid and ensemble approaches of VGG16 with other architectures enhance the generalization, regularization tools like dropout and batch normalization reduce overfitting^7^. These characteristics make VGG16 an ideal backbone for practical plant disease detection system.

### Interpretability with Grad-CAM

Model interpretability has become an essential factor for the real-life deployment of DL in agriculture. Gradient-influenced Class Activation Mapping (Grad-CAM) improves interpretability with heatmap representation that highlights the symptomatic region(s) affecting the prediction most significantly^16,17^. This ensures that biologically sound decisions are made and also establishes confidence among researchers^18,19^. Beyond credibility, Grad-CAM can also assist researchers in recognizing biases and adjusting model architectures^20^. When combined with powerful network architectures like VGG16 and ResNet, Grad-CAM can be used to design strong AI frameworks for plant disease detection^17^.

### Integration of AI with digital tools and web applications

The integration of AI with digital tools is transforming precision agriculture. IoT-based sensors when integrated with AI models provide the analysis on ongoing data stream for initiating real time field-oriented interventions^21,22^; Jadesha et al.^3^,. Web-based Decisions Support Systems provide enhanced capabilities to the farmers through predictive advisories, input use efficiency and risk decrease^23^; Jadesha et al.^3^,. These will enhance productivity and lower labour costs, while ensuring sustainable use of soil, water, and pesticides^24–26^; Jadesha et al.^3^,. They are scalable and automated thus can be applied to small- and large-scale farming^27,28^. While recent studies demonstrate the feasibility of integrating AI-based diagnostics into digital platforms, most implementations remain at a prototype or proof-of-concept stage, focusing on system integration rather than introducing new algorithmic methodologies. Accordingly, the present study is positioned as an applied and integrative investigation that prioritizes system-level validation, interpretability, and real-world deployment over the development of novel deep-learning architectures. In this context, the present work emphasizes applied system development and validation, aiming to bridge AI-based disease detection, interpretability, and field-level decision support under realistic constraints, rather than proposing novel learning architectures.

### Limitations and research gaps

Despite these advances, several significant challenges still remain to prevent the widespread application of AI in the identification and detection of plant diseases. There is a lack of properly annotated datasets that are also predominantly heterogeneous and cannot be used with the required general correction for a wide range of crops and agro-ecological zones^29^. Interpretability, though improved by Grad-CAM, remains a concern as many DL models are still perceived as “black boxes.” Environmental variability including fluctuating light, background noise, and diverse symptom expression further complicates reliable performance in field conditions^30^. The integration of AI-driven systems with IoT-enabled sensors and web-based advisory platforms is still underdeveloped, constraining practical deployment^21,22^. Addressing these gaps requires not only methodological advances but also carefully designed applied frameworks that explicitly acknowledge real-world constraints related to data availability, environmental variability, and user adoption, while progressively improving robustness through iterative field validation.

Accordingly, the novelty of this study lies in the integration, validation, and deployment of established AI techniques within a decision support framework, rather than in the development of new model architectures. Taken together, the convergence of DL architectures, interpretability tools, and digital platforms highlights the potential for integrated, farmer-friendly solutions for maize disease detection and management. The present study is positioned as an applied, integrative investigation, combining established AI techniques with field validation and digital deployment to assess feasibility and practical utility under current data and operational constraints. Against this backdrop, the present study was undertaken as an applied system-validation effort with the following objectives (i) AI-Based Disease Classification: to evaluate ML and DL models for SCLB detection in maize, focusing on classification accuracy, confusion matrix analysis, learning curves, and error/variance-based metrics. (ii) Model Interpretability and Visualization: To enhance biological interpretability of the VGG16 model using t-SNE feature visualization and Grad-CAM heatmaps. (iii) Fungicidal Field Evaluation: To assess the efficacy of fungicidal treatments against SCLB, analysing their effects on disease severity, grain yield, and profitability. (iv) Digital Advisory Development: To develop a farmer-friendly web application integrating AI-based detection with field findings for real-time SCLB advisory.

## Materials and methods

**Fig. 1:**
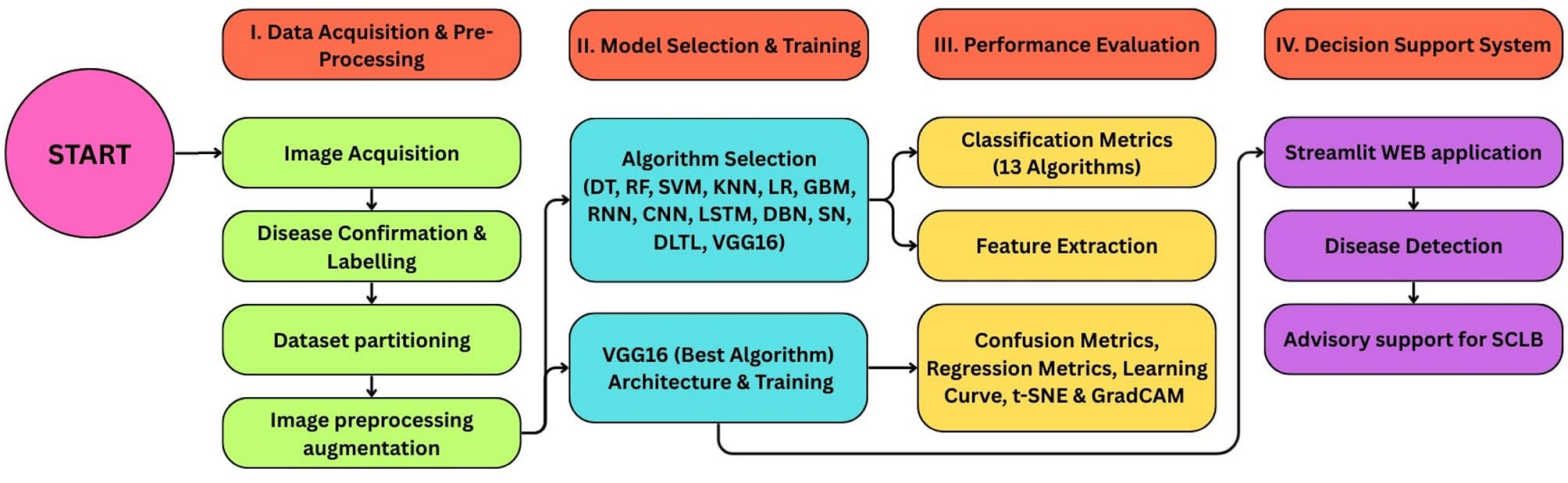
Methodological framework for SCLB diagnosis and farm advisory.

The methodological framework employed in this study is illustrated in Fig. 1, depicting the systematic workflow for SCLB diagnosis using ML and DL approaches.

The methodology encompasses sequential steps of (I) data acquisition and pre-processing, (II) Model selection and Training, (III) Performance evaluation, (IV) Decision Support System. Each component is described in detail below.

### Dataset acquisition and pre-processing

#### Image acquisition

Maize leaf images representing both healthy and Southern Corn Leaf Blight (SCLB)–infected specimens were systematically collected under a range of realistic field conditions, along with selected controlled environments, to support dataset representativeness. Field photographs were acquired from commercial maize farms, research plots, and greenhouse facilities across multiple agro-climatic zones where natural SCLB infections caused by *Bipolaris maydis* were observed and confirmed through in-field pathology assessments.

Images were captured using high-resolution digital cameras (minimum 12 MP) following standardized acquisition protocols to maintain consistent image quality while minimizing background interference. Data collection was conducted across different crop growth stages and naturally varying illumination conditions commonly encountered in field settings. While deliberate efforts were made to capture variability in lighting and canopy background, the dataset primarily reflects conditions feasible during routine field scouting and does not exhaustively represent all extreme illumination, canopy density, or disease severity scenarios.

The final dataset comprised images collected from our own field trials, including 1034 healthy and 1674 diseased samples, providing a reasonably balanced class distribution to mitigate class imbalance during model training. We note that further expansion of the dataset across additional seasons, locations, and environmental conditions would further enhance generalizability and is planned as part of future work. Accordingly, the evaluated model performance reflects image conditions with reasonable resolution, adequate leaf visibility, and limited occlusion, as typically achievable during guided field scouting.

#### Dataset splitting and augmentation strategy

Images used in this study were collected from independent maize plots, including research plots and farmers’ fields, representing multiple inbred lines and hybrids across geographically distinct locations in Karnataka and Himachal Pradesh, India. Each image corresponds to an independent plant observation, and no repeated images of the same leaf or plant were included.

Dataset partitioning into training, validation, and test sets was performed prior to data augmentation. Data augmentation techniques were applied exclusively to the training set to enhance model robustness, while the validation and test sets comprised only original, non-augmented images. This strategy minimized the risk of data leakage and ensured unbiased evaluation of model performance. A summary of the dataset composition and the use of data augmentation is provided in Supplementary Table S1.

#### Disease confirmation and labelling

Plant pathologists and certified disease specialists manually annotated all images following established SCLB diagnostic protocols. Microscopic examination was conducted to confirm the presence of *Bipolaris maydis* conidia and characteristic disease symptoms including elongated necrotic lesions with yellow halos. Standardized rating measures were used to evaluate the severity of the disease, and symptoms were categorized according to the size, shape, and distribution of lesions on the leaf surfaces. Quality control measures included double-blind validation by independent experts to ensure labelling accuracy. Images exhibiting ambiguous symptoms or poor quality were excluded from the dataset. The labelling process incorporated binary classification (healthy vs. diseased) to support comprehensive model training and evaluation. Although disease severity was recorded during field assessment, the image dataset used for model training and evaluation was intentionally labelled for binary classification (healthy vs. SCLB-infected) to support robust early-stage detection.

#### Dataset partitioning

The dataset was partitioned into training (70%), validation (15%), and test (15%) sets using stratified sampling to maintain consistent class distributions across subsets. The training set was used for model learning, the validation set for hyperparameter tuning and model selection, and the test set for unbiased final performance evaluation.

#### Image pre-processing and augmentation

To improve model robustness and standardize the dataset, image pre-processing techniques were used. To retain optimal computational efficiency and assure compatibility with Deep-learning architectures, all images were scaled to 224 × 224 pixels. To provide reliable training convergence, min-max scaling was used to standardize pixel values to the range. Numerous data augmentation approaches, such as random rotation (0–360° in 30° increments), flipping horizontally and vertically, brightness correction (± 20%), and contrast enhancement (0.8–1.2×), were used to expand the dataset size and strengthen the model’s generalization capabilities. All augmentation operations were applied exclusively to the training set, while validation and test images were retained in their original, non-augmented form.

### Model selection and training

#### Algorithm selection and implementation

Thirteen distinct machine-learning and deep-learning algorithms were systematically evaluated for SCLB classification to identify optimal approaches. Traditional machine-learning methods included Decision Trees, Random Forest, Support Vector Machines, K-Nearest Neighbors, Logistic Regression, Gradient Boosting Machines, and XGBoost. Advanced neural network architectures comprised Artificial Neural Networks with multiple hidden layers, Convolutional Neural Networks, Long Short-Term Memory networks, Deep Belief Networks, and Siamese Networks along with Deep-learning approaches with transfer learning included VGG16 were used.

#### VGG16 architecture implementation

The VGG16 architecture was selected as the primary Deep-learning model based on its proven effectiveness in plant disease classification tasks. The pre-trained VGG16 model, originally trained on ImageNet dataset, was fine-tuned using transfer learning techniques specifically for SCLB detection. The architecture consisted of 13 convolutional layers with 3 × 3 filters, 5 max-pooling layers with 2 × 2 windows, and 3 fully connected layers with dropout regularization (*p* = 0.5). Rectified Linear Unit (ReLU) activation functions were employed in hidden layers to introduce non-linearity, while the final output layer utilized softmax activation for probability distribution computation. Batch normalization was implemented between convolutional layers enhancing training speed and stability.

#### Software environment and implementation

The experimental framework was implemented using Python 3.8.10 with specialized libraries for ML and DL applications. TensorFlow 2.6.0 and Keras 2.6.0 provided the primary Deep-learning framework, while PyTorch 1.9.0 was utilized for specific model implementations. OpenCV 4.5.3 handled image processing operations, and Scikit-learn 1.0.2 supported traditional ML algorithms and evaluation metrics. NumPy 1.21.0 and Pandas 1.3.3 facilitated numerical computations and data manipulation. Matplotlib 3.4.3 and Seaborn 0.11.2 were employed for visualization and statistical plotting.

#### Hyperparameter optimization

To optimize model performance, grid search was employed for hyperparameter tuning, wherein predefined combinations of hyperparameters were systematically evaluated to identify optimal model configurations. Adaptive learning rate adjustments using the Adam optimizer were applied to ensure stable convergence. Furthermore, batch normalization and dropout were incorporated to enhance generalization and reduce overfitting.

### Performance evaluation

#### Comparison of classification metrics

Model performance was evaluated using a combination of classification and regression-based metrics to ensure a robust and comprehensive assessment. For all thirteen algorithms, standard classification metrics were employed, including Accuracy, Precision, Recall, F1-Score, AUC-ROC, Specificity, and Negative Predictive Value (NPV). These metrics collectively provided insights into both overall predictive performance and class-wise discrimination, accounting for the trade-off between sensitivity and specificity.

Accuracy reflects the proportion of correctly predicted disease occurrences relative to total predictions, thereby quantifying overall predictive correctness^31^. Precision, defined by Powers^32^, measures the fraction of true positive predictions among all positive predictions, indicating the reliability of positive classifications and minimizing false positives. Recall, also described by Powers^32^, quantifies the proportion of true positives identified among all actual positives, reflecting the model’s capacity to capture disease instances. To balance these measures, the F1-Score was employed, representing the harmonic mean of precision and recall and providing a single robust indicator of classification performance^33^. In addition, AUC-ROC was used to evaluate discriminative power across varying thresholds, while Specificity (true negative rate) and NPV offered complementary insights into the models’ ability to correctly identify and exclude healthy samples. A confusion matrix was also generated for each model, summarizing prediction outcomes against actual observations for a detailed performance breakdown^31^.

#### Comparison of feature extraction

Feature extraction was performed to measure the capability of different ML and DL algorithms to capture discriminative patterns from maize leaf images affected by SCLB. All input images were pre-processed through resizing and normalization to ensure consistency in scale and pixel distribution before being used for feature extraction. For DL models, particularly CNN, features were derived from the final convolutional or fully connected layers, while traditional ML algorithms utilized handcrafted feature representations generated from the pre-processed images.

To maintain fairness, the same dataset split was applied across all models, ensuring consistent evaluation of their feature extraction competences. This approach enabled a relative analysis of representational strength across ML and DL methods, highlighting their efficacy in distinguishing healthy from diseased samples. Including feature extraction analysis in the methodology provided a benchmark for model selection and interpretability, confirming that the most robust and biologically meaningful features were leveraged for downstream classification tasks.

#### Regression metrics analysis of VGG 16

For the most effective deep-learning model (VGG16), additional regression-based metrics were computed to assess predictive performance at the probability level. These metrics included Mean Absolute Error (MAE), Mean Squared Error (MSE), Root Mean Squared Error (RMSE), coefficient of determination (R²), and Explained Variance.

Although the task is formulated as a binary classification problem, these regression-based metrics were calculated using the predicted class probabilities generated by the VGG16 model rather than discrete class labels. This approach enabled quantification of the deviation between predicted probabilities and the corresponding ground-truth binary labels, thereby providing complementary insights into error magnitude, prediction consistency, and variance explanation.

Lower MAE, MSE, and RMSE values indicate closer alignment between predicted probabilities and true labels, while higher R² and Explained Variance values reflect improved consistency in probability-level predictions. These metrics were interpreted alongside standard classification metrics (accuracy, precision, recall, F1 score, and AUC) and were not intended to replace classification-based evaluation.

#### Learning curve analysis of VGG 16

To evaluate how model performance varies with training set size, a learning-curve analysis was performed for the VGG16 classifier and the best-performing traditional machine-learning models. The training data were incrementally sampled in 10% steps from 10% to 100% of the total training set, while the validation set remained fixed. For each subset, the models were trained using the established training pipeline, and the corresponding training and validation performance values produced during each run were collected automatically by the framework. These values were plotted against the proportion of training data using Matplotlib 3.4.3 to generate the learning-curve graphs.

#### t-SNE visualization of VGG 16

The VGG16 model’s trained high-dimensional feature representations were shown using t-distributed Stochastic Neighbor Embedding (t-SNE) analysis. To produce two-dimensional visuals that demonstrated the model’s ability to differentiate between healthy and SCLB-infected maize samples, deep features were taken from the network’s last layer and processed using t-SNE. It was possible to determine if healthy and diseased samples formed separate, separable clusters in the feature space by using color-coded markers to denote different classes in the generated scatter plots. To verify the model’s learning efficacy, cluster quality was assessed using common metrics. Well-separated clusters showed that VGG16 effectively recorded significant disease-specific patterns that could consistently distinguish between healthy and diseased maize leaves.

#### Grad-CAM analysis of VGG 16

Gradient-weighted Class Activation Mapping (Grad-CAM) was implemented to provide interpretable visual explanations of the VGG16 model’s decision-making process. This technique generated class-discriminative localization maps that highlighted the most important image regions influencing classification decisions by analysing gradient information from the final convolutional layer. The resulting heatmaps were overlaid onto original maize leaf images using color-coded intensity maps, where high-intensity regions (red/yellow) indicated areas strongly associated with SCLB disease symptoms and low-intensity regions (blue/green) represented areas with minimal diagnostic relevance. The visualization process ensured that heatmaps matched original image dimensions for accurate interpretation, while quantitative analysis measured the percentage of highly activated pixels to assess how precisely the model focused on disease-affected areas. This approach provided biological validation by confirming that the model’s attention was directed toward actual symptom locations rather than irrelevant background features, thereby increasing confidence in the automated diagnostic system’s reliability for real-world applications.

### Decision support system development using VGG 16

**Fig. 2:**
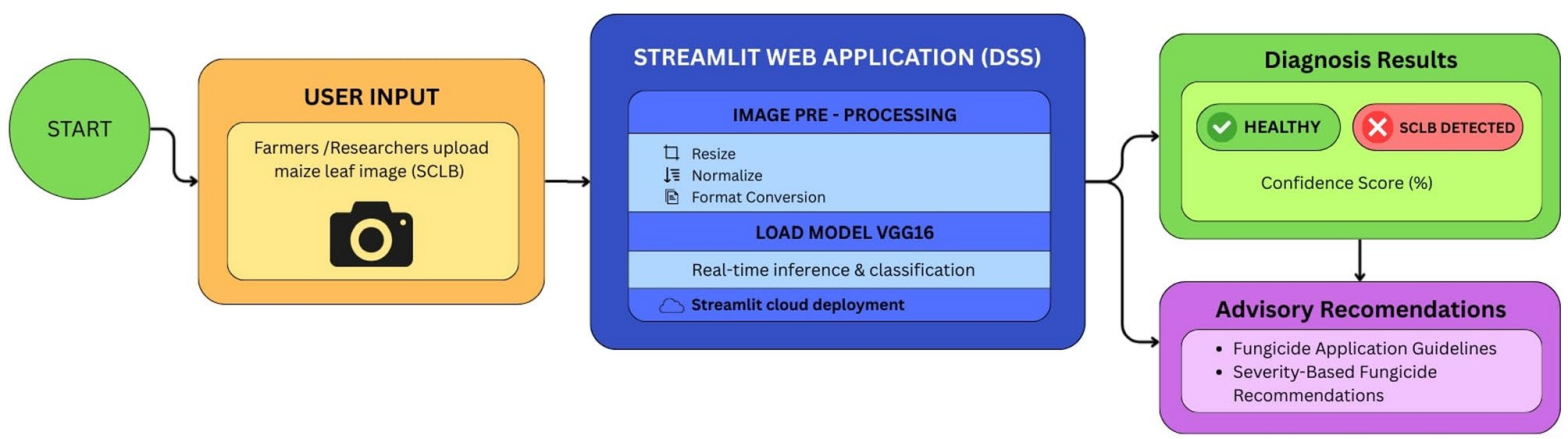
Decision support system architecture using VGG16 for SCLB detection.

A Decision Support System (DSS) was developed using the Streamlit framework, chosen for its lightweight deployment, real-time response, and user-friendly design (Fig. 2). The web-based platform is intended for farmers, agricultural extension workers, and researchers, providing a simple interface for uploading maize leaf images to determine disease status. Once an image is uploaded, it undergoes a series of automated pre-processing steps such as resizing, normalization, and format conversion to ensure compatibility with the fine-tuned VGG16 model trained on SCLB datasets. These steps help maintain consistency and accuracy across a wide range of input images. The processed images are then analysed by the VGG16 model, which performs real-time inference to classify each sample as either healthy or SCLB-infected. To enhance transparency, the system also generates confidence scores that indicate the reliability of each prediction. The results are displayed through a clear, user-friendly interface. Beyond diagnosis, the DSS also provides practical advisory recommendations, including fungicide application guidelines and preventive agronomic practices, enabling timely and effective management of SCLB in maize. The fungicide advisory provided by the DSS is derived from independently conducted field trials and is presented as a static, evidence-based recommendation; it is not dynamically optimized or modified by the AI model during deployment.

### Evaluation of fungicides against Southern Corn Leaf Blight

#### Experimental site, seed material, and fungicides

Field experiments were conducted during *kharif* 2023 and 2024 at the Maize Research Plots, Zonal Agricultural Research Station (ZARS), V.C. Farm, Mandya (12.56º N, 76.82º E; 603 m above MSL). The site is known to be highly prone to SCLB. The soil type was red loamy with recorded pH and EC values. The susceptible maize hybrid HT-5402 was used for the trials. Six commercially available fungicides were procured from the local market, with details provided in Table 1.

Sowing and treatment application: The field was prepared to a fine tilth, and seeds of HT-5402 were sown in rows at a spacing of 60 × 20 cm. Standard agronomic practices were followed to raise the crop. The experiment was laid out in a Randomized Complete Block Design (RCBD) with three replications. Each replication consisted of six rows, each 4 m in length. Fungicide treatments were applied using a knapsack sprayer at a spray volume of 500 L/ha. A single foliar spray was imposed at the first appearance of disease symptoms.

**Table 1:** Fungicides evaluated for the management of SCLB.

Fungicide	F	Trade name	Manufacturer	Target site	FRAC code	Type of fungicide	Fungicide group	Product rate/ha
Mancozeb	WP	Indofil M 45	Indofil Pvt Ltd	MSC	M03	Contact	Dithiocarbamates	2000 g
Kresoxim methyl	SC	Ergon	Tata Pvt Ltd	QOI	11	Systemic	Strobilurin	500 ml
Carbendazim + Mancozeb	WP	SAAF	UPL Pvt Ltd	TB + MSC	1 + 11	Combi	Benzimidazole + Dithiocarbamate	1000 g
Azoxystrobin + Difenoconazole	SC	Amistar Top	Syngenta Pvt Ltd	QoI + SBI	11 + 3	Combi	Strobilurin + Triazole	500 ml
Azoxystrobin + Cyproconazole	SC	AmpectXtra	Syngenta Pvt Ltd	QoI + SBI	11 + 3	Combi	Strobilurin + Triazole	500 ml
Pyraclostrobin + Epoxiconazole	SE	Opera	BASF Pvt Ltd	QoI + SBI	11 + 3	Combi	Strobilurin + Triazole	750 ml

F- Formulation, WP- Wettable Powder, SC- Suspension Concentrate, SE- Suspension emulsion, MSC-Multisite contact, SBI-Sterol Biosynthesis inhibitor, QoI-Quinone outside inhibitors, TB- tubulin polymerization.

Recording of observations: Disease incidence was recorded in each replication prior to fungicide application using the 1–9 disease severity scale^34^. The Percent Disease Index (PDI) was calculated following Wheeler^35^ using the formula: PDI = Sum of numerical ratings/(total number of leaves observed × Maximum grade). Data were analysed by ANOVA using SAS 9.4 software and multiple comparison tests were conducted using Turkey’s HSD (*p* ≤ 0.05) test.

## Results

### Machine-learning and deep-learning algorithms in SCLB disease detection

**Fig. 3:**
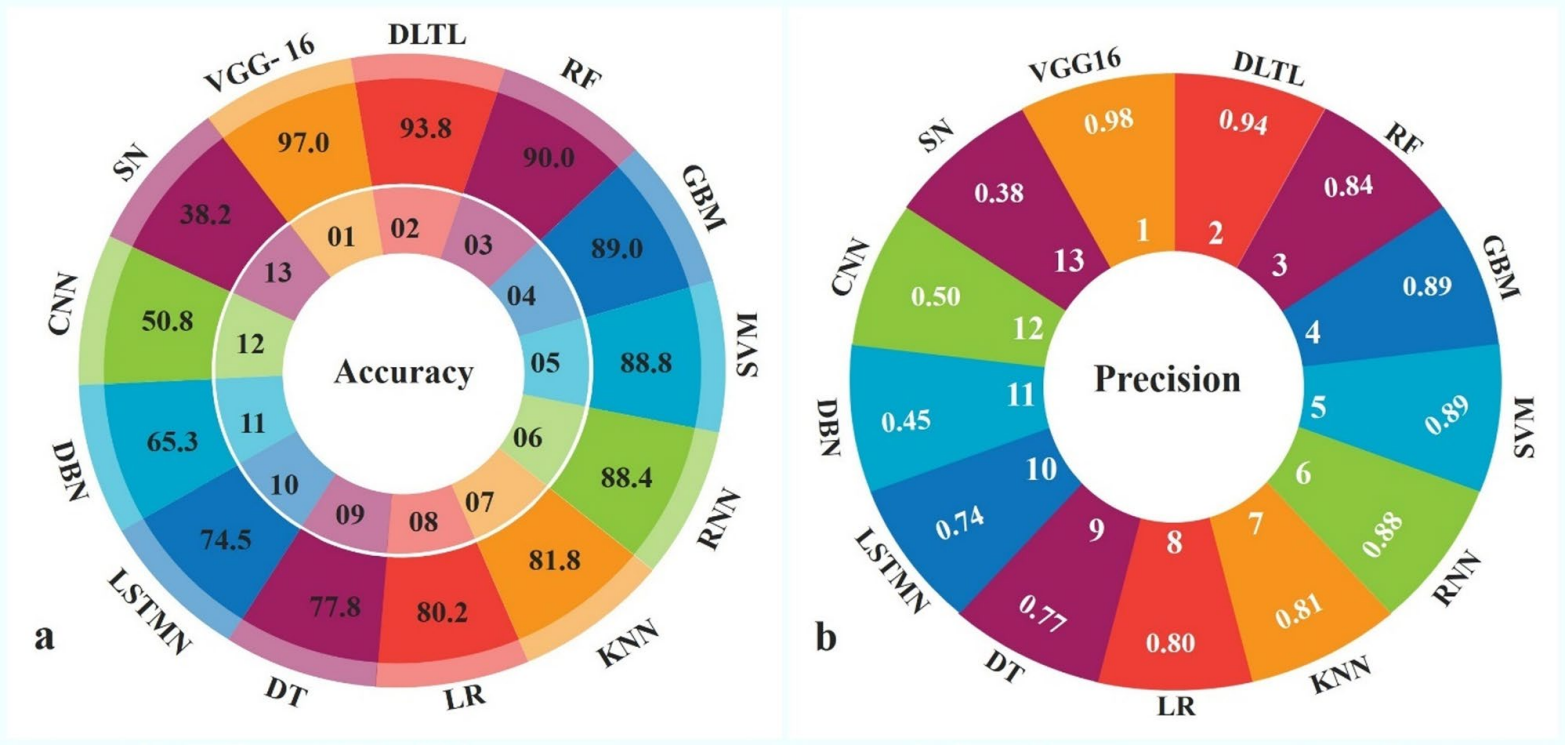
Accuracy and precision of algorithms in SCLB disease detection.

**Fig. 4:**
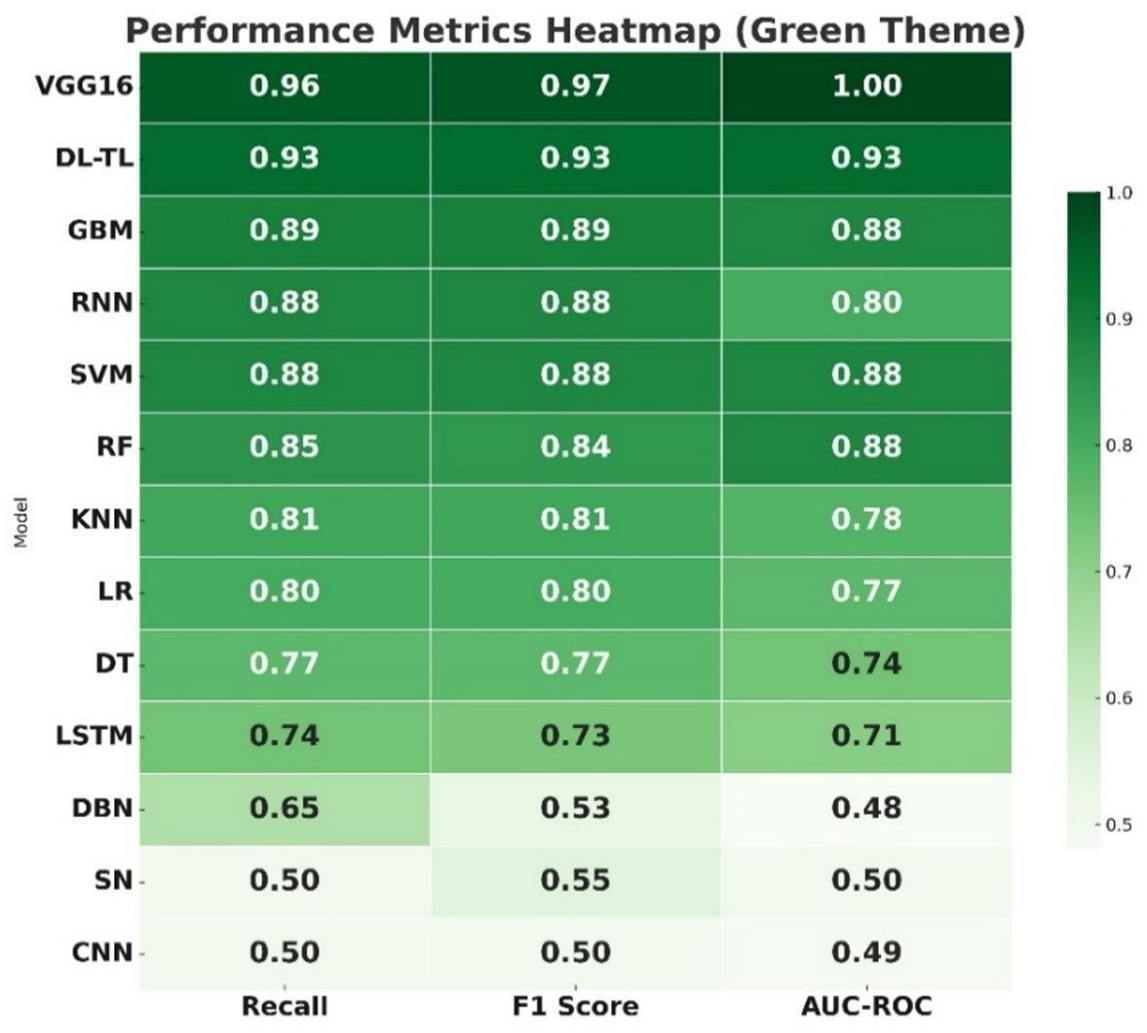
Recall, F1-score, and AUC–ROC of algorithms in SCLB disease detection.

The performance of thirteen machine-learning and Deep-learning algorithms was assessed for the detection of SLCB in maize. The results revealed considerable variation in accuracy and other evaluation metrics across the algorithms (Figs. 3 and 4).

Among the conventional algorithms, Decision Trees achieved 77.8% accuracy with balanced precision, F1 score and recall (0.77 each). Logistic Regression (80.2%) and K-Nearest Neighbors (81.8%) performed slightly better, while Support Vector Machines (88.8%) and Gradient Boosting Machines (89.0%) stood out as the best performers among the traditional ML approaches, showing strong balance across all metrics with AUC values of 0.88 each. Random Forest also performed robustly with 90.0% accuracy, though its precision and recall were relatively lower (0.84 and 0.85, respectively). In the case of neural network architectures, Long Short-Term Memory networks reached 74.5% accuracy, surpassing Deep Belief Networks (65.3%) and Convolutional Neural Networks (50.8%). Recurrent Neural Networks performed substantially better with 88.4% accuracy, closely approaching the top ML algorithms. However, Siamese Networks exhibited poor performance with only 38.2% accuracy, precision of 0.38, and the lowest AUC (0.50).

Models using transfer learning and Deep-learning yielded a striking improvement. Transfer Learning based DL garnered 93.8% accuracy with impressive precision (0.94), recall (0.93) and F1 score (0.93) along with AUC being of performance: 0.93. Best overall performing was achieved by VGG16, 97.0% accuracy and a precision of 0.98, recall of 0.96 and an F1 score of 0.97. VGG16 also attained AUC of 1.00, indicating that it possessed a better discriminative ability in classifying the SCLB than other algorithms. While the VGG16 model demonstrated exceptionally high performance under the evaluated conditions, these results should be interpreted within the context of the dataset size, the binary disease classification framework, and the specific field acquisition conditions represented in this study. In general, Deep-learning methods, particularly transfer learning and VGG16 were significantly superior. All these emphasized the promise of powerful DL architectures for highly precise and dependable disease diagnosis in maize.

### Feature extraction scores of ML and DL algorithms for SCLB detection

**Fig. 5:**
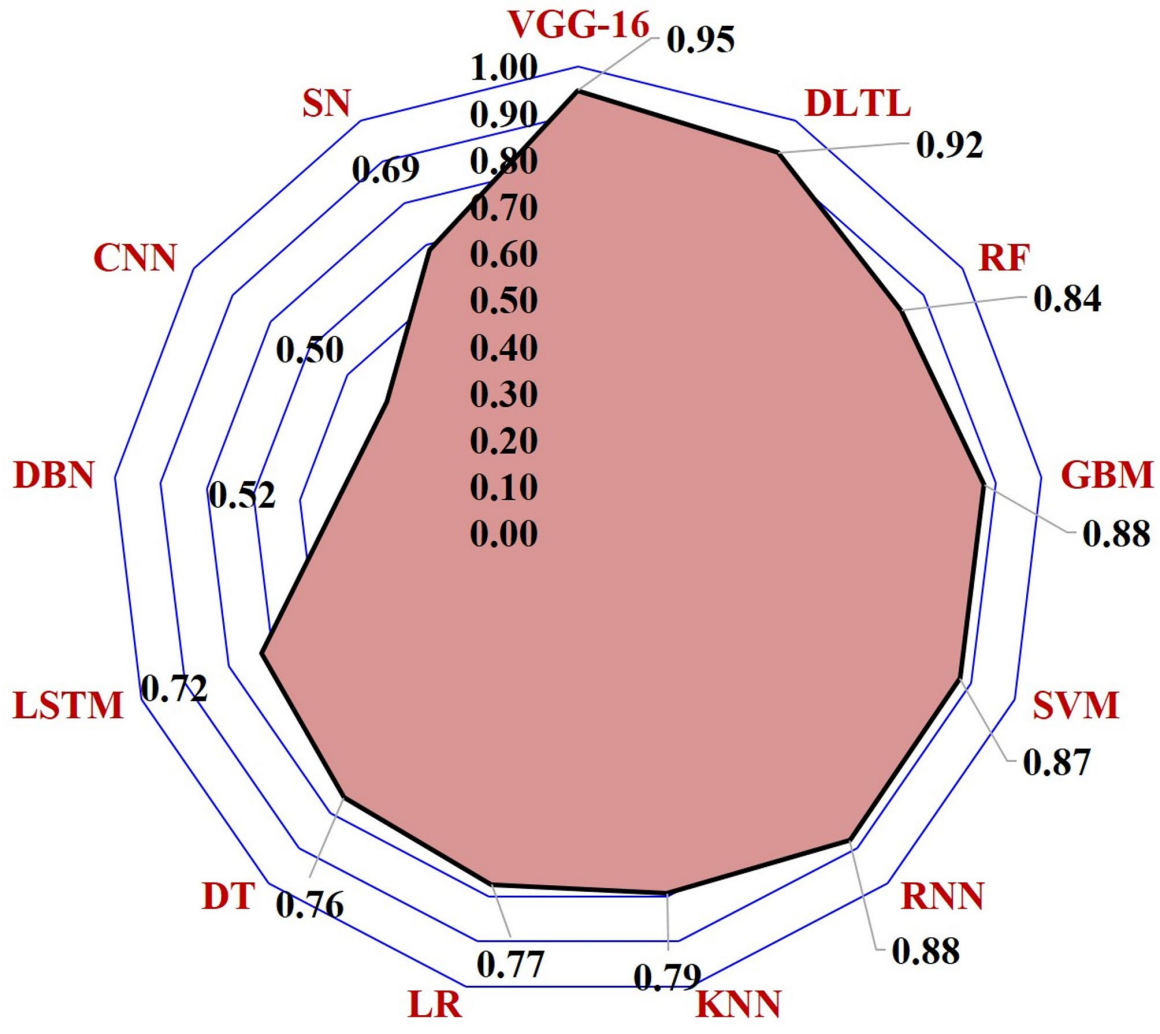
Comparative performance of algorithms in feature extraction from SCLB images.

Feature extraction scores were compared to assess the ML and DL algorithms to extract significant characteristics for the classification of SCLB (Fig. 5). With the highest feature extraction score (0.95) among all the algorithms, VGG16 demonstrated a significant capacity to learn deep hierarchical representations of images of both healthy and diseased maize leaves. The benefit of pre-trained networks in capturing discriminative disease traits was confirmed by Deep-learning with Transfer Learning (0.92), which came in close second.

Gradient Boosting Machines (0.88), Recurrent Neural Networks (0.88), and Support Vector Machines (0.87) were among the ensemble approaches that demonstrated competitive performance. Comparing Random Forest (0.84) to traditional machine-learning methods, the former showed a strong feature extraction capability. However, the feature extraction capabilities of K-Nearest Neighbors (0.79), Logistic Regression (0.77), and Decision Trees (0.76) were found to be modest. Long Short-Term Memory Networks (0.72), Siamese Networks (0.69), Deep Belief Networks (0.52), and Convolutional Neural Networks without transfer learning (0.50) all received lower scores, indicating that they were less effective at differentiating intricate visual patterns in the dataset.

Overall, the feature extraction results show the classification performance, with VGG16 and transfer learning-based DL algorithms consistently outperforming traditional ML and shallow DL algorithms in effectively capturing disease-specific features from maize leaf images.

### Error and variance-based performance assessment of VGG16

**Fig. 6:**
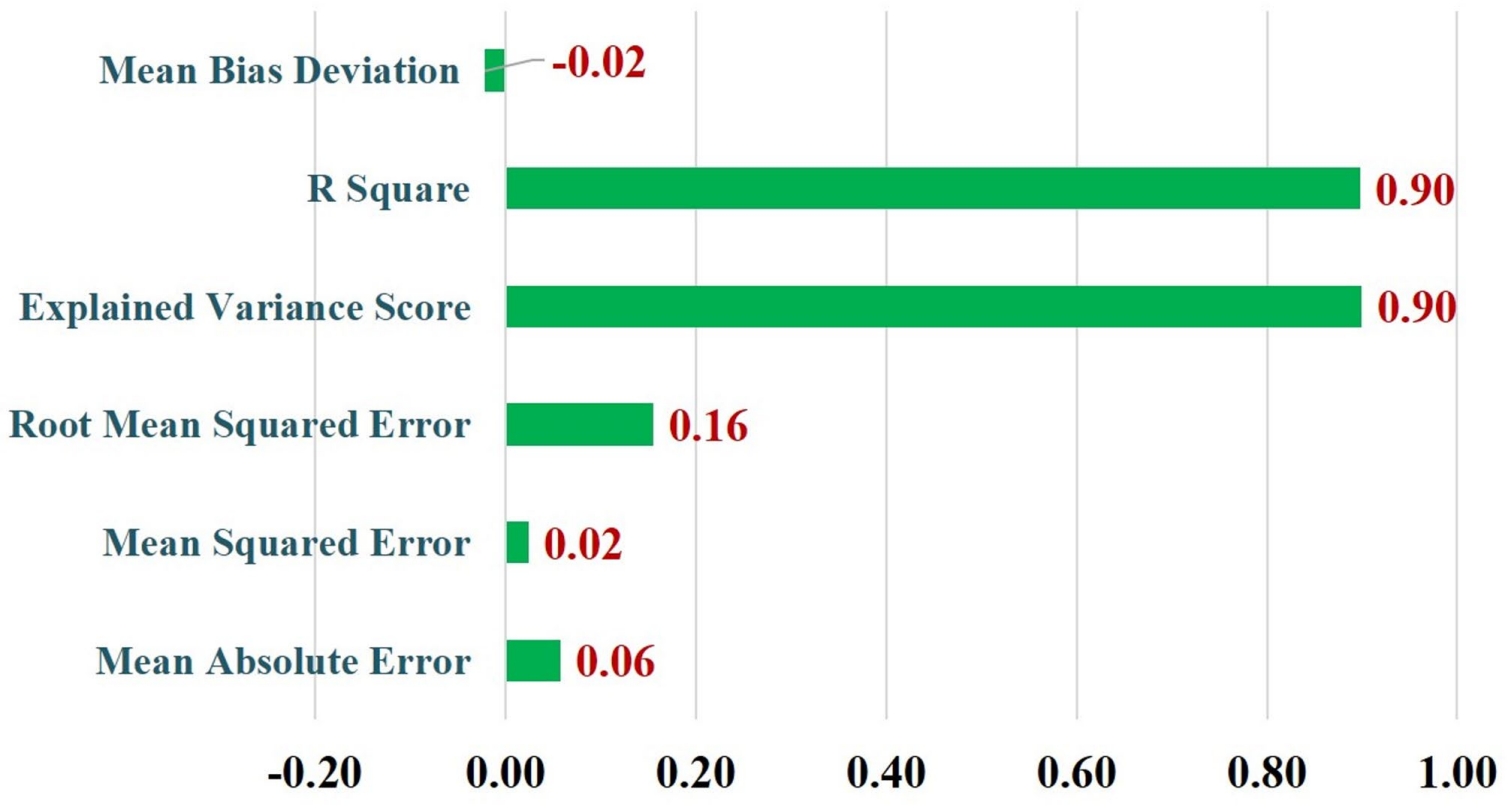
Error and variance-based performance assessment of VGG16 for SCLB detection.

Since VGG16 demonstrated the highest performance in classification metrics among all tested models, it was further evaluated using error-based and variance-based statistical measures (Fig. 6) for SCLB prediction. The model achieved a low Mean Absolute Error (MAE = 0.06) and Mean Squared Error (MSE = 0.02), with a corresponding Root Mean Squared Error (RMSE = 0.16), confirming minimal prediction errors. The Explained Variance Score (0.90) and R² Score (0.90) indicate that VGG16 accounted for 90% of the variance in disease classification, signifying a high level of reliability. Additionally, the Mean Bias Deviation (MBD = − 0.02), being very close to zero, reflects negligible bias in predictions. Collectively, these results reinforce the robustness of the VGG16 architecture, establishing it as the most accurate and consistent model for classifying SCLB disease in maize images.

### Confusion matrix analysis of VGG16 for SCLB detection

**Fig. 7:**
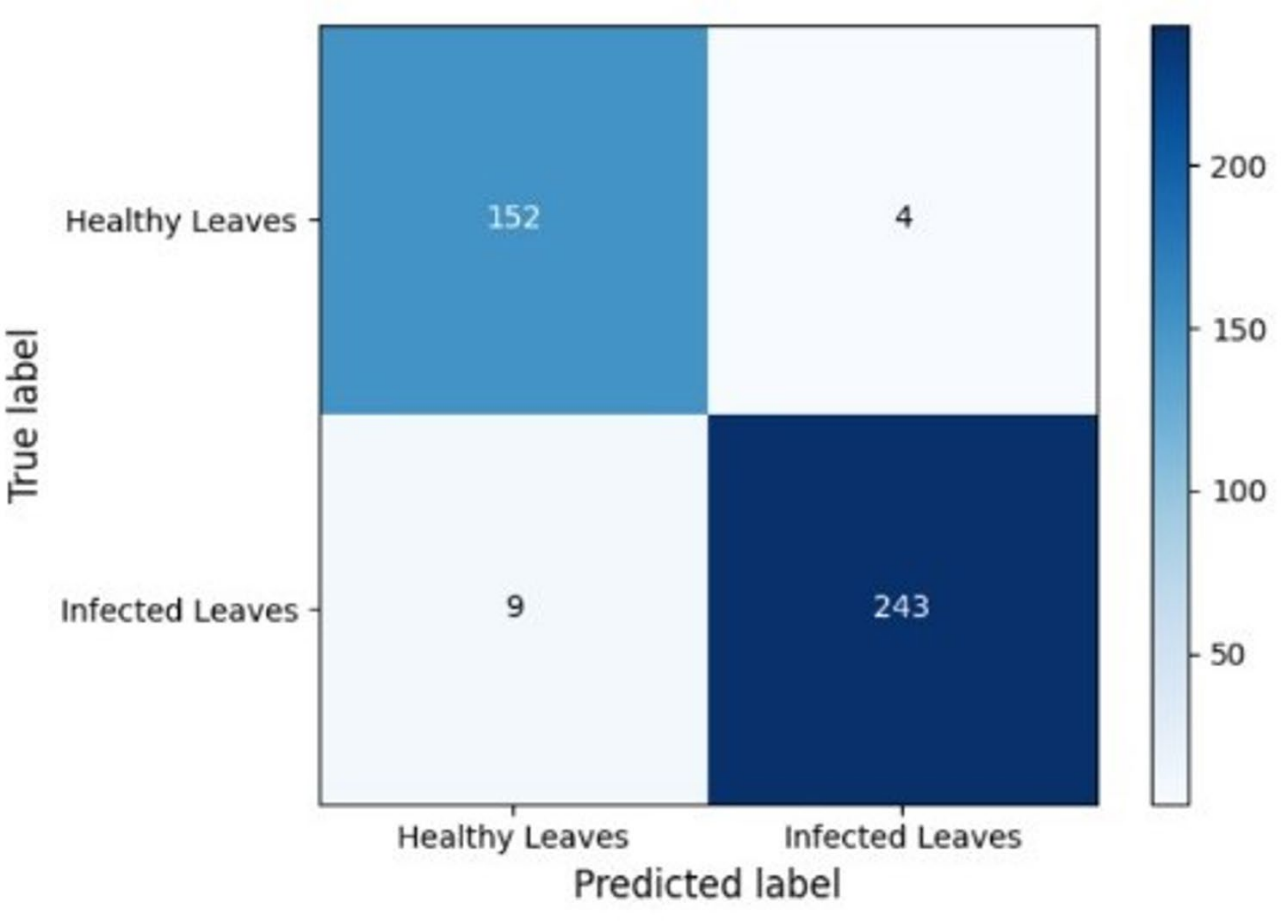
Confusion matrix analysis of VGG16 for SCLB detection.

To evaluate the prediction reliability of the top-performing algorithm, VGG16, for SCLB, its confusion matrix was examined (Fig. 7). Only four diseased leaves were misclassified as healthy (false negatives) and nine healthy leaves as diseased (false positives) by the algorithm, which properly identified 152 diseased samples and 243 healthy samples.

This demonstrated the VGG16 architecture’s resilience in differentiating between SCLB-infected and healthy maize leaves, as evidenced by the extremely high true positive rate (recall = 0.96) and true negative rate (specificity = 0.98). The model’s great generalization ability and usefulness for automated disease identification in real-world situations are demonstrated by the minimal number of misclassifications.

### Learning curve patterns of VGG16 model on SCLB dataset

**Fig. 8:**
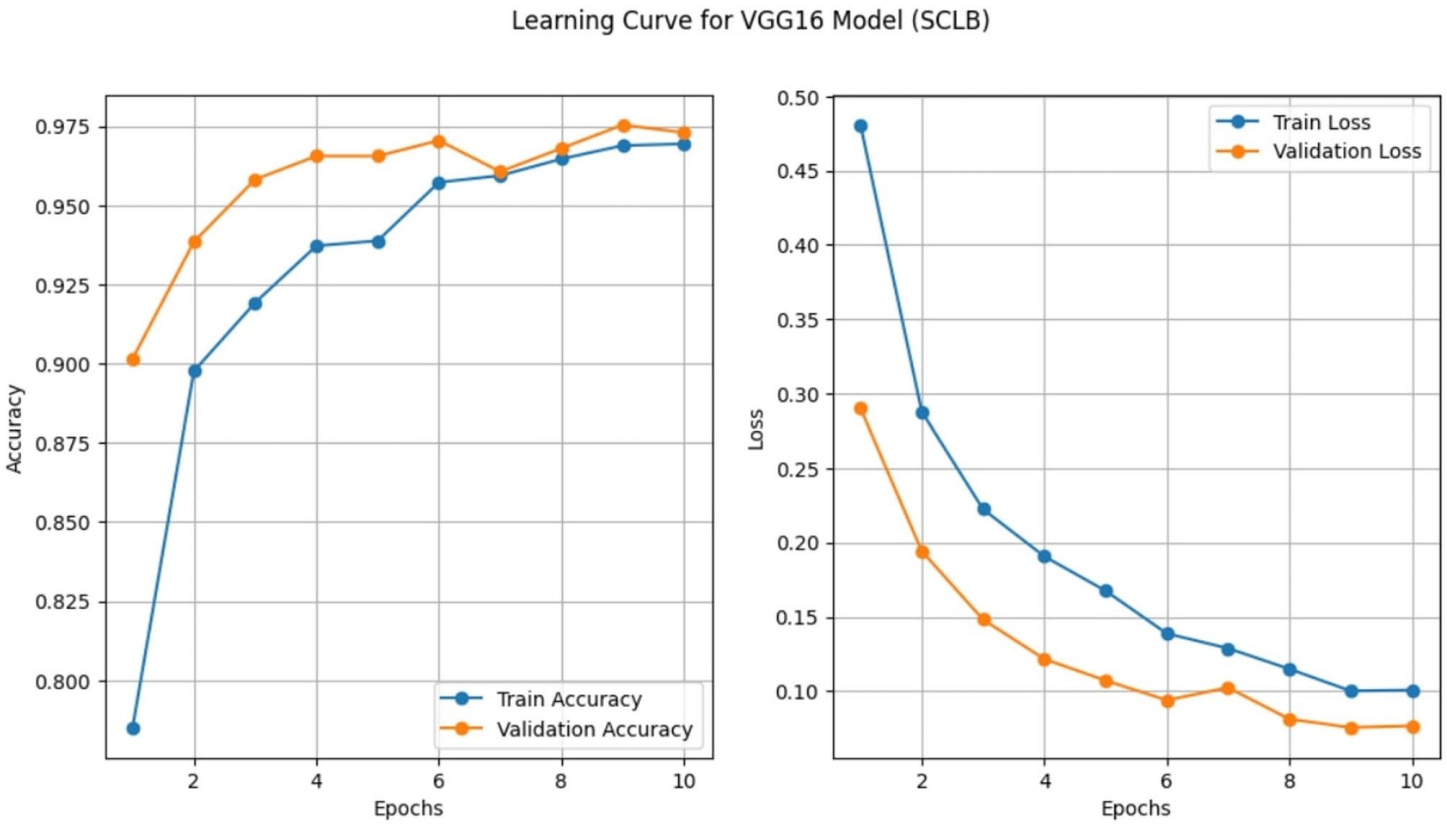
Learning curve patterns of VGG16 in SCLB detection.

Figure 8 displays the VGG16 model’s learning curves for SCLB detection. With the number of epochs, the training and validation accuracies (Fig. 8, left) improved continuously until the tenth epoch, when they were 0.97 for training and 0.975 for validation.

This suggests that strong discriminative characteristics could be learned by the model without overfitting. Likewise, with every epoch, the training and validation losses (Fig. 8, right) steadily dropped. The model’s smooth convergence and consistent generalization ability were demonstrated by the training loss decreasing from 0.48 to 0.10 and the validation loss decreasing from 0.29 to 0.08. The tight match between the training and validation curves demonstrates that VGG16 was not significantly overfitted or underfitted, making it an extremely dependable architecture for in maize SCLB detection.

### t-SNE visualization of feature representations by VGG16 for SCLB disease

**Fig. 9:**
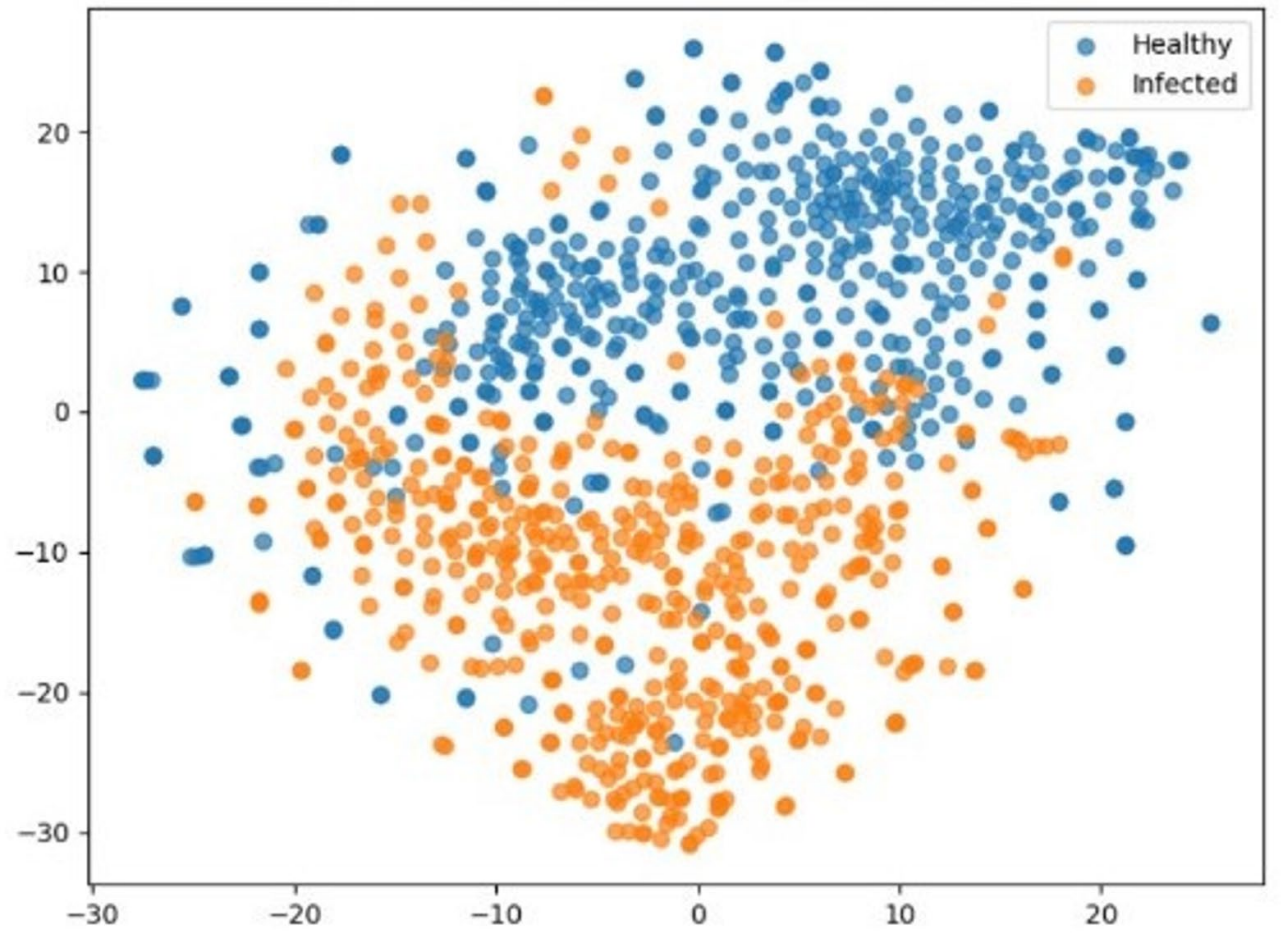
t-SNE visualization of feature representations generated by VGG16.

A t-distributed Stochastic Neighbor Embedding (t-SNE) analysis was conducted to assess the discriminative potential of the deep features that were recovered by the top-performing model, VGG16 (Fig. 9).

The 2D display showed that the clusters of SCLB-infected (orange) and healthy (blue) maize leaves were clearly distinguished. VGG16’s high capacity to capture disease-specific traits was demonstrated by the fact that most infected samples were categorized apart from healthy samples. The cluster boundaries showed a slight degree of overlap, which is consistent with the few misclassifications (false positives and false negatives) shown in the confusion matrix. This finding supports VGG16’s robustness for precise and trustworthy SCLB illness detection by demonstrating that it not only learnt highly separable feature representations but also attained superior classification metrics.

### Grad-CAM based interpretability of VGG16 for SCLB detection

**Fig. 10:**
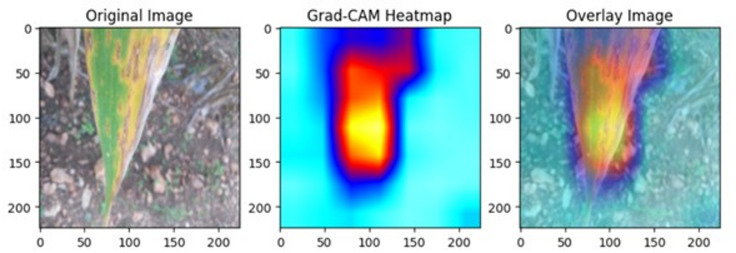
Grad-CAM–based interpretability of VGG16 in SCLB detection.

Gradient-weighted Class Activation Mapping (Grad-CAM) was used to show the areas of maize leaves that contributed most to the classification decision in order to improve the interpretability of the VGG16 model (Fig. 10).

The Grad-CAM heatmap (centre) depicts the areas of maximum activation, whereas the original image (left) displays the characteristic symptoms of SCLB. The overlay image (right) demonstrates that VGG16 correctly targeted leaf symptoms, including lengthy necrotic lesions with yellow haloes that are typical of SCLB. By showing that the model’s predictions were based on disease-relevant traits rather than background noise, this visualization offers biological interpretability. This explainability boosts confidence when using the model for precision agriculture and real-world disease diagnosis applications.

### Field evaluation of fungicides against SCLB in maize

**Fig. 11:**
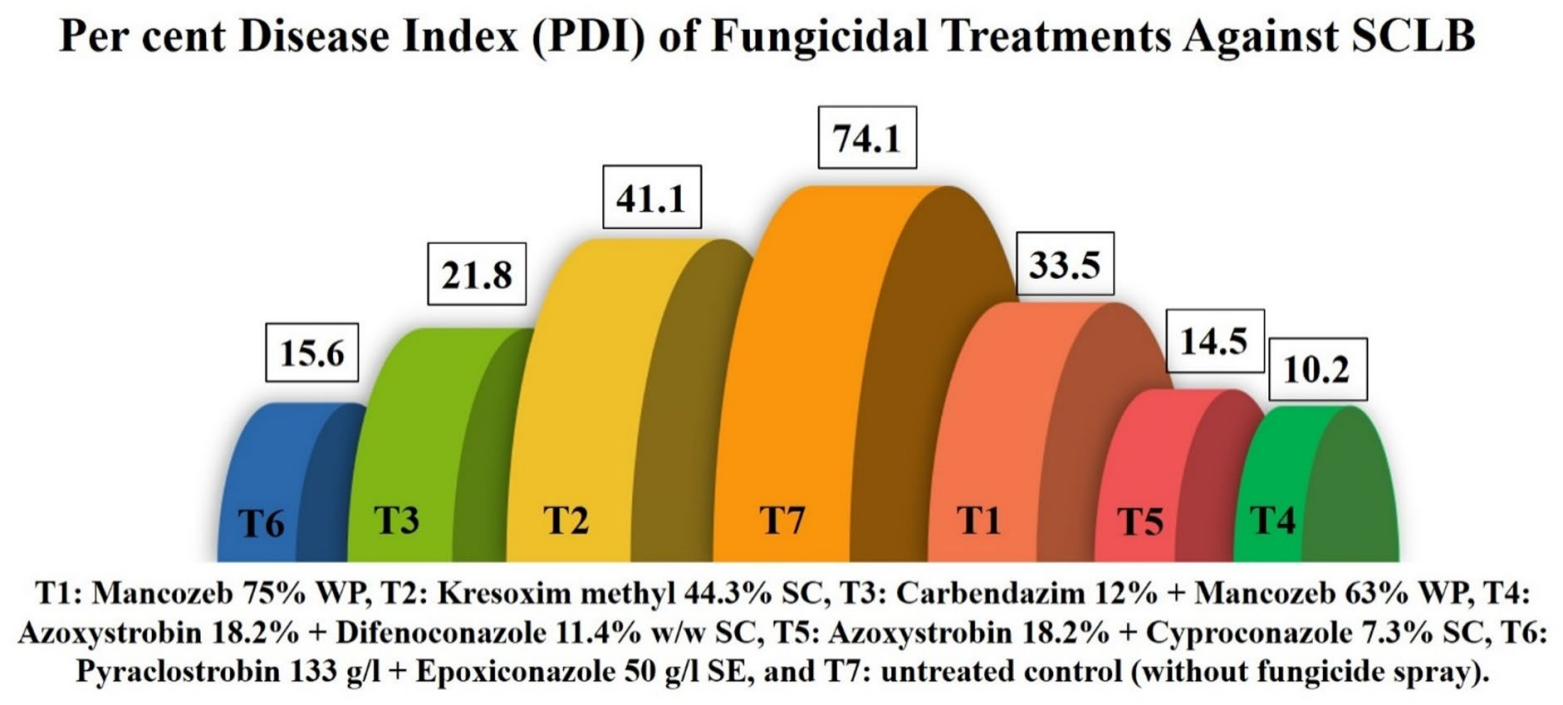
Field evaluation of fungicides against SCLB.

Fungicide efficacy against Southern Corn Leaf Blight was evaluated through independent field trials conducted over two consecutive seasons using standard agronomic protocols, and these evaluations were performed independently of the AI-based disease detection framework. Field evaluation of fungicides revealed significant differences in their efficacy against SCLB as measured by Percent Disease Index (PDI) and percent reduction over control (PROC) (Fig. 11 & Table 2).

**Table 2:** Comparative field evaluation of fungicides on SCLB percent disease index (PDI) during 2023–2024.

Fungicides	PDI of SCLB during 2023				PDI of SCLB during 2024				Mean PROC
	Before Spray	15 DAS	30 DAS	PROC	Before Spray	15 DAS	30 DAS	PROC	
Mancozeb75%WP	13.3	29.3	34.4	53.2	14.1	26.7	32.5	56.5	54.9
Kresoxim methyl 44.3% SC	16.6	33.4	39.7	45.9	15.7	31.9	42.4	43.2	44.5
Carbendazim 12% + Mancozeb 63% WP	15.7	17.5	19.9	72.9	14.9	16.5	23.6	68.4	70.7
Azoxystrobin 18.2% + Difenoconazole 11.4% w/w SC	14.9	8.9	9.7	86.7	15.5	10.1	10.6	85.8	86.2
Azoxystrobin 18.2% + Cyproconazole 7.3% SC	15.3	12.1	13.1	82.1	13.6	11.0	15.8	78.8	80.5
Pyraclostrobin 133 g/l + Epoxiconazole 50 g/l SE	17.2	11.2	14.2	80.7	15.6	8.7	16.9	77.4	79.0
Control	15.4	51.7	73.4	-	14.5	53.4	74.7	-	-
CD(*p* = 0.05)		1.48	2.23			1.12	7.64		
SEm±		0.48	0.72			0.36	2.48		
CV (%)		3.56	4.3			2.78	13.88		

T. No -Treatment Number, PDI- Percent Disease Index, DAS-Day after spray, PROC-Percent reduction over control.

With PDI values rising from about 15% prior to spray to over 70% at 30 days after spraying (DAS), the disease spread quickly in the untreated control plots in both years, demonstrating the strong disease pressure in the field. Azoxystrobin 18.2% + Difenoconazole 11.4% SC had the highest mean PROC of 86.2% and the lowest PDI values (9.7% and 10.6% at 30 DAS in 2023 and 2024, respectively) among the fungicidal treatments, suggesting excellent and reliable protection. This was closely followed by Azoxystrobin 18.2% + Cyproconazole 7.3% SC (mean PROC 80.5%) and Pyraclostrobin 133 g/l + Epoxiconazole 50 g/l SE (mean PROC 79.0%), both of which also maintained considerably lower PDI compared to the control. The fungicide combination Carbendazim 12% + Mancozeb 63% WP exhibited moderate efficacy, with mean PROC of 70.7%. Although it reduced disease severity compared to untreated plots,

its performance was relatively lower than that of strobilurin-triazole mixtures. Single active fungicides such as Mancozeb 75% WP (54.9% PROC) and Kresoxim methyl 44.3% SC (44.5% PROC) provided the least protection, with higher PDIs at 30 DAS, indicating their reduced effectiveness under the prevailing field conditions. Overall, the results clearly demonstrated that fungicidal mixtures containing strobilurins and triazoles were most effective in suppressing SCLB, with Azoxystrobin + Difenoconazole emerging as the most promising treatment for disease management.

### Cohen’s d based comparative evaluation of fungicides

The efficacy of different fungicides against SCLB in maize was evaluated, and their performance was compared with the untreated control (74.05). Substantial reductions in disease intensity were observed in all fungicidal treatments, which was further confirmed by Cohen’s d effect size analysis (Table 3).

**Table 3:** Cohen’s d–based comparative evaluation of fungicides for SCLB management.

Treatment	Cohen’s d vs. Control	Interpretation
Mancozeb 75% WP	−1.82	Very large effect – strong reduction compared to control
Kresoxim methyl 44.3% SC	−1.48	Large effect – notable reduction compared to control
Carbendazim 12% + Mancozeb 63% WP	−2.34	Very large effect – stronger reduction compared to control
Azoxystrobin 18.2% + Difenoconazole 11.4% SC	−2.86	Extremely large effect – strongest reduction among all treatments
Azoxystrobin 18.2% + Cyproconazole 7.3% SC	−2.67	Extremely large effect
Pyraclostrobin 133 g/l + Epoxiconazole 50 g/l SE	−2.61	Extremely large effect

Interpretation of Cohen’s d values: 0.2 = small effect, 0.5 = medium effect, 0.8 = large effect, and > 1.5 = very large effect.

Among the fungicides, Azoxystrobin 18.2% + Difenoconazole 11.4% SC recorded the lowest disease intensity (10.15), showing the highest effect size (Cohen’s d = −2.86), indicating an extremely large effect compared to control. Similarly, Azoxystrobin 18.2% + Cyproconazole 7.3% SC (14.45; d = −2.67) and Pyraclostrobin 133 g/l + Epoxiconazole 50 g/l SE (15.55; d = −2.61) exhibited extremely large effects, reflecting their superior efficacy in SCLB management. The combination fungicide Carbendazim 12% + Mancozeb 63% WP (21.75; d = −2.34) also showed a very large effect in reducing disease severity. Mancozeb 75% WP (33.45; d = −1.82) and Kresoxim methyl 44.3% SC (41.05; d = −1.48) were comparatively less effective but still demonstrated large to very large effects. Overall, the results revealed that all tested fungicides significantly reduced SCLB severity compared to the untreated control, with the triazole-strobilurin combinations proving to be the most effective in managing the disease.

### Effect of fungicidal treatments on grain yield and profitability of maize under SCLB

Significant differences in grain yield of maize were observed among the fungicidal treatments (Table 4). The untreated control recorded the lowest yields (65.8 q/ha in 2023 and 62.3 q/ha in 2024; mean 64.1 q/ha), reflecting the severe impact of SCLB disease on crop productivity. Among the fungicidal treatments, the highest yields were obtained with Azoxystrobin 18.2% + Difenoconazole 11.4% SC, which produced 87.1 q/ha in 2023 and 80.3 q/ha in 2024, with a mean yield of 83.7 q/ha. This treatment also recorded the highest per cent increase over control (PIOC) of 30.4% and the most favourable cost-benefit ratio (1:2.41). The combinations Azoxystrobin 18.2% + Cyproconazole 7.3% SC and Pyraclostrobin 133 g/l + Epoxiconazole 50 g/l SE followed closely, with mean yields of 82.0 q/ha and 80.5 q/ha, mean PIOC of 27.9% and 25.7%, and CB ratios of 1:2.35 and 1:2.34, respectively. These treatments were statistically on par with Azoxystrobin + Difenoconazole. Moderate yield improvement was observed with Carbendazim 12% + Mancozeb 63% WP, which produced a mean yield of 75.0 q/ha (PIOC 17.0%) and a CB ratio of 1:2.20, indicating partial disease suppression and economic benefit. Single fungicides such as Mancozeb 75% WP (mean yield 72.1 q/ha, PIOC 12.5%, CB ratio 1:2.11) and Kresoxim methyl 44.3% SC (mean yield 66.6 q/ha, PIOC 4.1%, CB ratio 1:1.82) were comparatively less effective in improving yields. Overall, fungicidal mixtures of strobilurins and triazoles significantly enhanced maize yield and profitability, with Azoxystrobin + Difenoconazole proving most effective, followed by Azoxystrobin + Cyproconazole and Pyraclostrobin + Epoxiconazole.

**Table 4:** Comparative performance of fungicides on maize yield during Kharif 2023 and 2024.

T. No.	Fungicides	Kharif 2023		Kharif 2024		Mean		CB ratio
		Yield (q/ha)	PIOC	Yield (q/ha)	PIOC	Yield (q/ha)	PIOC	
T1	Mancozeb75%WP	75.2	14.3	69.0	10.8	72.1^**bc**^	12.5	1:2.11
T2	Kresoxim methyl 44.3% SC	70.5	7.1	62.7	1.0	66.6^**bc**^	4.1	1:1.82
T3	Carbendazim 12%+ Mancozeb 63% WP	77.8	18.2	72.3	16.1	75.0^**b**^	17.0	1:2.20
T4	Azoxystrobin 18.2% + Difenoconazole 11.4% w/w SC	87.1	32.4	80.3	28.7	83.7^**a**^	30.4	1:2.41
T5	Azoxystrobin 18.2% + Cyproconazole 7.3% SC	85.7	30.2	78.3	25.7	82.0^**a**^	27.9	1:2.35
T6	Pyraclostrobin 133 g/l + Epoxiconazole 50 g/l SE	82.4	25.2	78.7	26.7	80.5^**ab**^	25.7	1:2.34
T7	Untreated control (UTC)	65.8		62.3		64.1^**d**^		1:1.82
	CD (5%)	9.0		9.8		6.4		
	SEm	4.2		4.6		3.1		
	CV	9.4		11.4		10.4		

PIOC-Percent Increase Over control, CB= Cost Benefit Ratio.

## Discussion

### Comparative performance of ML and DL algorithms

While VGG16 is an established convolutional neural network, its selection in this study was driven by its robustness and suitability for field deployment, with the novelty residing in comprehensive evaluation, interpretability analysis, and integration into a decision support system rather than architectural modification. The accurate and early detection of Southern Corn Leaf Blight in maize is a cornerstone for minimizing yield losses and enhancing crop management strategies. A comparison of machine-learning and Deep-learning algorithms in this work showed that DL models especially those based on transfer learning, such VGG16 performed noticeably better than conventional ML algorithms in the diagnosis of SCLB disease. While traditional machine-learning techniques like random forests, decision trees, and support vector machines could identify modest disease patterns, they had trouble processing complicated, high-dimensional visual data. This drawback is consistent with other research showing that ML models frequently rely on manually created features, which makes it difficult to identify subtle, disease-specific cues in plant photos^11,12^.

In contrast, Deep-learning methods are able to detect fine-grained lesion characteristics by automatically learning hierarchical representations from raw image data. Specifically, the model was able to improve performance on comparatively smaller agricultural image sets by utilizing pre-trained weights on large-scale datasets through transfer learning with VGG16. Such capability has been repeatedly emphasized in previous studies, where pre-trained networks showed superior generalization and robustness compared to standalone ML models (Turkoglu and Hanbay, 2019; Zhu et al.^9^,. Our results add evidence that DL frameworks are not only computationally superior but also biologically relevant, as they effectively discriminate between healthy and diseased maize leaves across diverse field conditions. VGG16 emerged as effective feature extraction from SCLB lesions, leading to high classification accuracy and stable performance. These observations are also supported by recent research. For instance, VGG16 has been used in conjunction with hybrid or ensemble learning techniques to classify plant diseases with accuracies of above 95%^9^. High generalization over a range of ambient conditions was also shown in investigations that integrated VGG16 with Vision Transformers (ViTs) (Ahmad et al., 2023; Tabbakh and Barpanda^15^,. Our results support these conclusions, showing that using VGG16 for transfer learning is not only a methodological decision but also a useful step toward reliable plant disease diagnosis.

### Learning curves and regression-based evaluation

The learning curve analysis indicated that VGG16 consistently learned robust discriminative features without overfitting, as evidenced by smooth convergence of training and validation losses. This suggests strong generalization capacity and applicability to real-world field conditions. Importantly, we extended the evaluation beyond classification metrics to include regression-based measures such as MAE, MSE, RMSE, and Coefficient of Determination. These metrics offered deeper insights into error magnitude and variance explanation, thereby validating the reliability of VGG16 predictions.

Few plant disease classification studies report such comprehensive evaluations, making this an important contribution. By complementing classification metrics with regression-based assessments, we provided a multidimensional understanding of model robustness, consistency, and predictive power. Such rigorous evaluation is essential for translating AI models from experimental setups to practical agricultural applications^36^.

### Interpretability through Grad-CAM visualization

A major limitation of DL models has been their “black box” nature, raising concerns about interpretability and trust. In this regard, our study’s incorporation of Gradient-weighted Class Activation Mapping yielded insightful results. The biological significance of the model’s predictions was confirmed by Grad-CAM heatmaps, which consistently revealed disease-relevant regions, such as light-brown, circular to elongated necrotic lesions with dark edges. This interpretability not only enhanced transparency but also bridged the gap between computational predictions and plant pathology expertise.

Comparable studies have highlighted the importance of visual interpretability for adoption in agriculture. Prince et al.^17,18^ emphasized that Grad-CAM enhances user trust by showing that predictions are driven by symptom-relevant features rather than irrelevant background noise. Our results corroborate this and extend its utility by demonstrating that Grad-CAM can also be a model debugging tool. For instance, heatmaps enable researchers to detect biases, such as the model focusing on leaf edges or shadows, and subsequently refine datasets or training protocols. Grad-CAM thus not only improves interpretability but also accelerates iterative model improvement.

### Integration with web applications for Real-World deployment

**Fig. 12:**
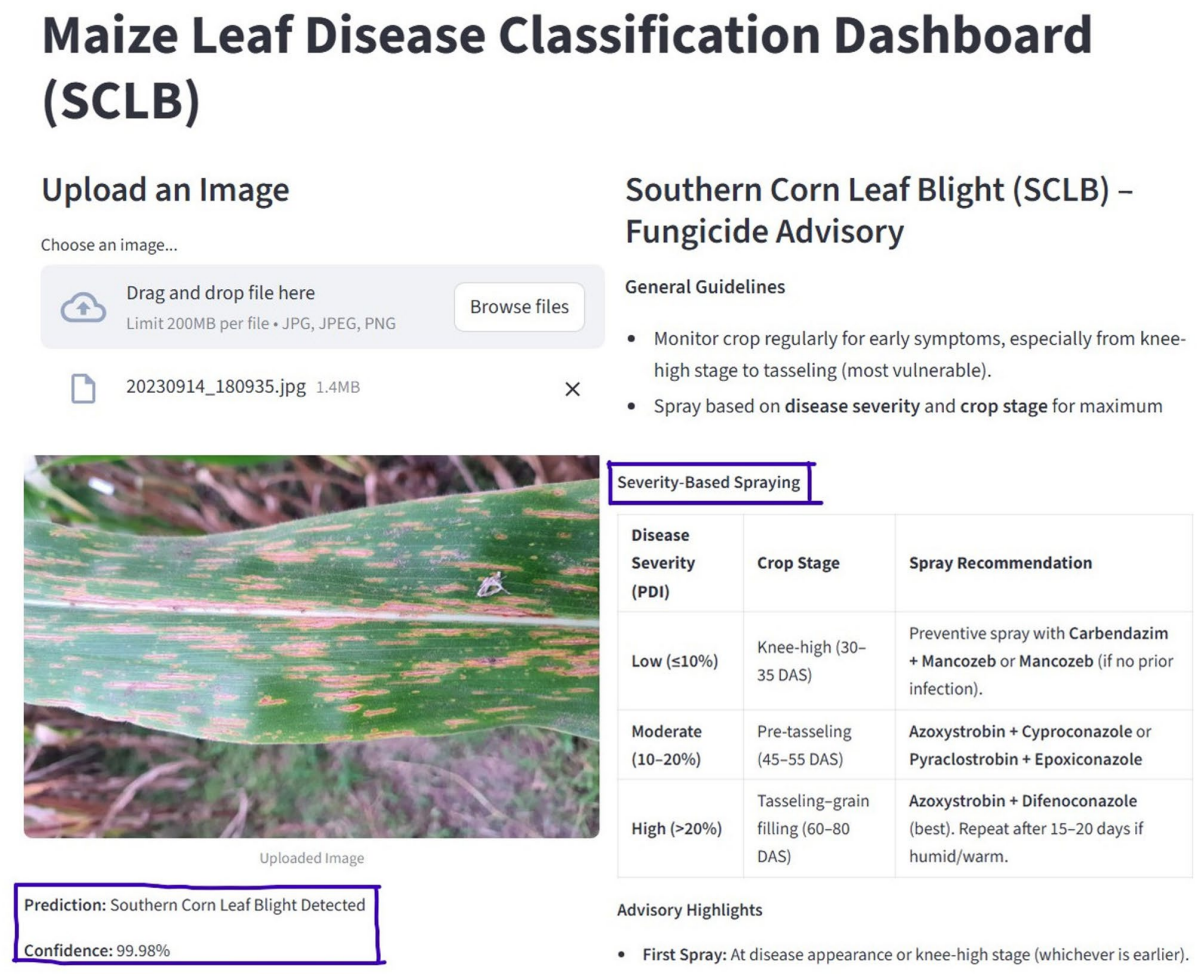
Final decision support system for SCLB diagnosis and farm advisory.

Although model correctness is crucial, its full potential is revealed when it is included into platforms that are easy to use and accessible. Precision agriculture has taken a revolutionary stride with the integration of our VGG16-based classifier with a web application. Real-time disease monitoring, farmer advising services, and scalable implementation across various farming systems are made possible by web-based technologies (Fig. 12). When combined with these applications, IoT-enabled sensors can provide time-sensitive and location-specific data for the best possible illness treatment (Jadesha et al.^3^,. The viability of farmer-centred disease detection systems has been proven by established digital agriculture platforms like Plantix. However, by combining disease-specific (SCLB) advising services with Grad-CAM interpretability, our method offers a unique benefit. Visually illustrating the disease regions on maize leaves not only improves diagnostic accuracy but also boosts researcher and farmer confidence. Furthermore, farmers in remote locations may now manage diseases thanks to AI-driven web tools that lessen reliance on field-level specialists. However, challenges such as limited digital literacy, uneven smartphone accessibility, unreliable internet connectivity, and the cost of smart devices remain significant barriers to adoption, particularly for smallholder farmers. Addressing these constraints will require simplified user interfaces, multilingual support, offline or low-bandwidth functionality, and close integration with existing agricultural extension systems. Importantly, the AI-driven diagnostic module and the fungicide recommendation component operate independently, ensuring that treatment advisories are grounded in validated agronomic evidence rather than automated optimization.

For broad acceptance, these socio-technical obstacles must be removed. To guarantee that digital agriculture solutions are both technologically sophisticated and socially inclusive, cooperation between researchers, extension agents, and legislators is necessary.

### Broader implications for precision agriculture

For sustainable agriculture, the combination of DL, interpretability tools, and web-based advising systems has significant ramifications. First, precise disease identification minimizes production costs and environmental effects by reducing the needless use of fungicides (Jadesha et al.^3^,; Hossain et al.^37^,. Second, prompt advice systems help farmers implement precision management techniques, which boost output and lower crop losses. Third, AI-based frameworks for disease control are in line with the global objectives of sustainable intensification, which place a high value on environmental stewardship and resource efficiency (Jadesha et al.^3^,; Hossain et al.^37^,.

### Research gaps and future directions

Although our results show that DL models can be integrated with digital advising platforms, there are still a number of unanswered questions. Larger and more varied annotated datasets that capture differences across agro-climatic zones, growth phases, and co-occurring stressors are the main obstacle. Generalization across regions is still restricted in the absence of such datasets. Future work will therefore also focus on improving model robustness under suboptimal image capture conditions commonly encountered in farmer-operated environments, through expanded field datasets, targeted data augmentation, and user-guided image acquisition protocols. Second, there is a pressing need to develop models capable of multi-disease and multi-label classification, as crops are frequently affected by multiple infections and stressors simultaneously rather than as discrete diseases. The development of multi-label classification frameworks that account for this complexity should therefore be a primary focus of future research. In particular, future work will focus on integrating disease severity estimation and simultaneous detection of multiple foliar diseases under real field conditions. Third, there are encouraging prospects for precision spraying and extensive disease surveillance when AI is combined with IoT, drones, and robotics. Field management may undergo a transformation thanks to the combination of real-time data from sensors and AI-powered advice systems. But doing so necessitates resolving issues with scale, cost, and data compatibility. Future research may explore AI-enabled optimization of fungicide recommendations by integrating real-time disease severity estimates, weather data, and adaptive feedback mechanisms, while maintaining agronomic and regulatory safeguards. Lastly, more work is required to improve model interpretability, both using Grad-CAM and by creating visualization and explanation tools that are easy for farmers to use. These initiatives will guarantee that AI systems continue to be open, reliable, and broadly usable. Future deployment efforts should prioritize farmer-centric design, capacity-building through extension networks, and hybrid advisory models that combine AI-based tools with human expertise to ensure inclusive and scalable adoption.

### Limitations of the study

Despite the promising results demonstrated in this study, several limitations should be acknowledged. First, the disease detection task was formulated as a binary classification problem (healthy vs. SCLB-infected), which does not capture intermediate disease severity levels or co-occurring foliar diseases that may be present under real field conditions. Consequently, disease severity grading, multi-disease classification, and mixed infection scenarios were beyond the scope of the present study. Extending the framework to multi-class or severity-aware classification would enhance practical applicability.

Second, although images were collected from geographically distinct locations, the dataset remains limited in terms of seasonal coverage, environmental diversity, and agro-ecological variability. Consequently, the reported performance reflects the evaluated conditions, and broader generalizability across additional regions, crop seasons, and management practices requires further validation using larger, multi-season datasets. In real-world farmer settings, variations in image quality arising from suboptimal lighting, partial leaf occlusion, camera motion, or background clutter may reduce diagnostic accuracy compared to controlled or guided image acquisition conditions.

Third, the performance of image-based AI models is inherently influenced by image quality, including factors such as illumination, focus, occlusion, and background complexity. In addition, the effectiveness of digital advisory tools depends on user-side constraints, including access to suitable imaging devices, network connectivity, and digital literacy among end users. These factors may affect real-world adoption and performance and should be carefully considered during large-scale deployment.

Future work will focus on addressing these limitations through expanded data collection, incorporation of disease severity estimation, robustness testing under extreme field conditions, and user-centered design approaches to improve accessibility and adoption.

## Conclusion

While established deep-learning architectures were employed, the study’s contribution is rooted in their applied integration with interpretability tools and independently validated field interventions, rather than algorithmic innovation. The present study demonstrates that integrating artificial intelligence with field-based disease management can significantly improve the precision management of Southern Corn Leaf Blight in maize. Among the thirteen evaluated algorithms, VGG16 emerged as the most reliable model, achieving superior classification accuracy, robust feature extraction, biological interpretability, and stability across error- and variance-based estimators. Field trials further validated that strobilurin-triazole fungicide combinations, particularly Azoxystrobin 18.2% + Difenoconazole 11.4% SC, effectively suppressed disease severity, enhanced grain yield, and provided the most favourable cost–benefit ratio. It is important to note that fungicide efficacy was validated through independent field experiments and was not directly optimized or influenced by the AI-based disease detection outputs. This further underscores the applied and validation-oriented nature of the proposed framework. Importantly, these findings were operationalized into a farmer-friendly web application that enables real-time disease diagnosis and field-level advisory services. Effective large-scale adoption of such digital advisory systems will, however, depend on addressing farmer digital literacy, smartphone accessibility, internet connectivity, and integration with existing agricultural extension networks, particularly in smallholder farming contexts. Collectively, the study establishes a holistic framework that combines explainable AI diagnostics, validated fungicidal strategies, and digital advisory tools, offering a scalable and sustainable model for managing SCLB and potentially other foliar diseases of maize. Nevertheless, the reported performance reflects the evaluated dataset, binary classification setting, and acquisition conditions, and further validation using larger, multi-season datasets across diverse agro-ecological zones and disease severity levels will be essential to assess broader generalizability.

## Supplementary Information

Below is the link to the electronic supplementary material.

Supplementary Material 1

## Data Availability

The datasets generated and/or analysed during the current study are available from the corresponding author on reasonable request.
