# Supplementary material for: Digital decision support integrated with diagnostics and precision fungicide application for Southern Corn Leaf Blight in maize
Source: Sci Rep. 2026 Feb 11;16:8217. doi: 10.1038/s41598-026-38151-0 (PMC12963633; doi:10.1038/s41598-026-38151-0)
Supplement: Supplementary file 1 — Supplementary Material 1 [file 41598_2026_38151_MOESM1_ESM.docx]

**Supplementary Table S1. Dataset composition and use of data augmentation**

| **Dataset subset** | **Healthy images** | **SCLB-infected images** | **Total images** | **Data augmentation applied** |
| --- | --- | --- | --- | --- |
| Original dataset | 1034 | 1674 | 2708 | No |
| Training set | Subset of original images | Subset of original images | — | Yes |
| Validation set | Subset of original images | Subset of original images | — | No |
| Test set | Subset of original images | Subset of original images | — | No |

Note: Data augmentation was applied exclusively to the training set to improve model robustness. All performance metrics reported in this study were computed using only the original, non-augmented images from the validation and test sets.
